# Annexin A5 Derived from Cell-free Fat Extract Attenuates Osteoarthritis via Macrophage Regulation

**DOI:** 10.7150/ijbs.92802

**Published:** 2024-05-19

**Authors:** Zhuoxuan Jia, Bijun Kang, Yushan Dong, Mingzhe Fan, Wei Li, Wenjie Zhang

**Affiliations:** 1 Department of Plastic and Reconstructive Surgery, Shanghai 9th People's Hospital, Shanghai Jiao Tong University School of Medicine, Shanghai Key Laboratory of Tissue Engineering, National Tissue Engineering Center of China, 639 ZhiZaoJu Road, Shanghai 200011, China.; 2 Shanghai Key Laboratory of Materials Laser Processing and Modification, School of Materials Science and Engineering, Shanghai Jiao Tong University, Shanghai, 200240, China.

**Keywords:** Osteoarthritis, Annexin A5, Macrophage

## Abstract

Osteoarthritis (OA) is a challenging degenerative joint disease to manage. Previous research has indicated that cell-free fat extract (CEFFE) may hold potential for OA treatment. This study investigated the role of Annexin A5 (AnxA5) within CEFFE in regulating macrophage polarization and protecting chondrocytes. *In vitro* experiments demonstrated that AnxA5 effectively inhibited M1 macrophage polarization by facilitating toll-like receptor (TLR) 4 internalization and lysosomal degradation through calcium-dependent endocytosis. This process decreased TLR4 expression, suppressed pro-inflammatory mediator release, and reduced the production of reactive oxygen species. Furthermore, AnxA5 displayed protective effects against chondrocyte necrosis and apoptosis. *In vivo,* studies revealed that intra-articular administration of AnxA5 ameliorated pain symptoms in a monosodium iodoacetate-induced osteoarthritis rat model. Histological analyses indicated a decrease in synovial inflammation and mitigation of cartilage damage following AnxA5 treatment. These results underscored the potential of AnxA5 as a therapeutic option for OA due to its capacity to regulate macrophage polarization and maintain chondrocyte viability. Further investigation into the specific mechanisms and clinical applications of AnxA5 may help improve the management of OA.

## Introduction

Osteoarthritis (OA), one of the most prevalent diseases affecting older people, is characterized by cartilage degeneration, subchondral remodeling, and synovial inflammation 1, 2. The exact pathogenesis of OA remains uncertain. However, mounting evidence supports the involvement of apoptosis in joint cartilage and activation of innate inflammatory pathways, mainly through synovial macrophages 3-6.

Our previous study demonstrated that cell-free fat extract (CEFFE), a cell-free liquid obtained mechanically from adipose tissue, may benefit OA treatment. Similar to the paracrine factors produced by adipose tissue-derived stem cells, CEFFE may help alleviate OA by suppressing synovial inflammation by regulating M1 macrophages 7. However, the complex composition of CEFFE, which includes over 1700 bioactive proteins, hinders its use as a clearly defined pharmaceutical ingredient suitable for large-scale production 8.

To address this challenge, we have shifted our focus toward identifying the essential components of CEFFE, thereby enabling recombinant protein technology to ensure quality and industrial scalability 9, 10. Through *in vitro* screening of macrophage proteins, Annexin A5 (AnxA5) has emerged as a potentially crucial protein. AnxA5, a Ca^2+^-dependent phospholipid-binding protein expressed in various tissues, plays diverse roles in intracellular signal transduction and vesicle transport 11, 12. It has been shown that AnxA5 can self-assemble into two-dimensional arrays on membranes upon Ca^2+^ activation, contributing to membrane repair and potentially reducing chondrocyte damage 13, 14.

This study aims to establish AnxA5 as a core immune-regulatory protein in CEFFE and validate its therapeutic efficacy in early OA by leveraging its cell-protective and macrophage-regulating properties. By elucidating the role of AnxA5 in OA, this study may pave the way for targeted protein therapies for this condition.

## Materials and methods

### Purification and identification of inhibitory factor of macrophage polarization in CEFFE

CEFFE was prepared as described in our previous study 7. In brief, after obtaining approval from the Ethics Committee of the Shanghai Ninth People's Hospital, adipose tissues were collected from the abdominal or thigh adipose tissues of 10 healthy female volunteers aged 22-35, following informed consent. The adipose tissues were washed with physiological saline to remove blood and debris, followed by centrifugation, mechanical emulsification, freezing at -80 °C, and rapid thawing of the middle-fat layer. Subsequent centrifugation yielded four layers after the freeze-thaw cycle. After filtering, the protein concentration in the third liquid layer was determined using a BCA kit (Sigma-Aldrich, USA) to obtain CEFFE.

**Figure 1:**
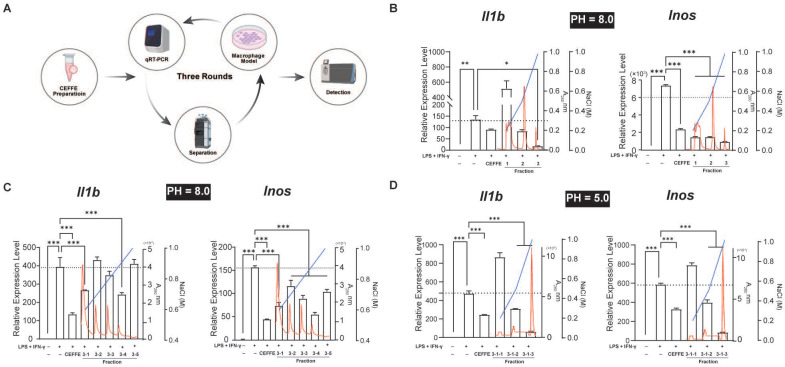
Profiling Macrophage Polarization Regulatory Proteins in CEFFE using an *in Vitro* Model. **A.** The schematic workflow for screening. **B**, **C**, and **D**. Three-stage ion exchange chromatography separation of CEFFE proteins and their impact on *in vitro* macrophage polarization and inflammatory mediator secretion (represented by black bars). Red line indicates absorbance at 280 nm, while blue line represents NaCl concentration, black dashed lines indicate the mRNA levels in the LPS+IFN-γ treated groups. Data presented as mean ± standard deviation (n = 3 per group). ^*^p < 0.033; ^**^p < 0.002; ^***^p < 0.001; ns, not significant.

CEFFE underwent three rounds of fractionation (Figure 1A). Initially, CEFFE was applied to a Q Focurose FF column (Huiyan Biology, China) with 40 mM sodium phosphate buffer, pH 8.0, and proteins were eluted using a gradient of 20%, 50%, and 100% 1 M NaCl and 40 mM sodium phosphate buffer, pH 8.0. Protein concentration was determined using by measuring absorbance at 280 nm. The impact of the fractionated conditioned media on macrophage polarization was accessed by directly adding each fraction to an *in vitro* macrophage polarization model. Specific experimental procedures can be found in our previous study 7. The initial concentration of CEFFE was 5 mg/mL. Following sample collection from each component, volumes were adjusted to match the input volume, and 100 μL/mL from each component was used for cell experiments. In the second round, proteins bound to the Q Focurose FF matrix were eluted with a gradient of 60%, 70%, 80%, 90%, and 100% 1 M NaCl and 40 mM sodium phosphate buffer, pH 8.0, following the same protocol as the first round. For the third round of fractionation, the buffer was switched to 40 mM sodium phosphate buffer, pH 5.0, and proteins bound to the Q Focurose FF matrix were eluted with a gradient of 20%, 50%, and 100% 1 M NaCl. Protein analysis was conducted using Liquid Chromatography Tandem-Mass Spectrometry (LC-MS/MS) after the final round of fractionation.

### Culture of mouse bone marrow-derived macrophages (BMDMs) and Raw264.7 cells

Primary BMDMs were isolated from male C57BL/6 mice (Weitong Lihua Biotechnology, China) aged 4-6 weeks. The mice were euthanized, and femurs and tibia were removed to extract BMDMs. These cells were obtained by flushing the bones with a cold culture medium, passing through a 70-μm cell strainer (Corning, USA), and centrifuging at 1500 rpm for 5 minutes. The isolated cells were cultured in a medium containing 20 ng/mL M-CSF (R&D Systems, USA) for 5 days. Medium replacement occurred on Day 3 and Day 5 before using the cells for experiments. The culture medium consisted of α-Minimum Essential Medium (MEM), 10% fetal bovine serum (FBS, #141c, Gibco) and 1% penicillin-streptomycin-gentamicin solution (Invitrogen, Thermo Fisher Scientific, USA). Mature BMDMs were identified via double-staining with flow cytometry antibodies CD16/32 (#101301, 1:200, BioLegend, USA) and F4/80 (#111602, 1:200, BioLegend, USA), which CD16/32^+^F4/80^+^ subsets exceeded 90% (Supplementary Figure 1A). Mouse macrophage Raw264.7 cells procured from the American Type Culture Collection cell bank (#TIB-71) were cultured in high-glucose Dulbecco's Modified Eagle Medium (DMEM, Invitrogen, Thermo Fisher Scientific, USA).

### In vitro polarization induction of BMDMs and Raw264.7 macrophages

The recombinant protein AnxA5 utilized in this research was obtained from SEME Cell Technology Co., Ltd (China). Raw264.7 cells were seeded at a density of 4 × 10^5^ in 6-well plates, whereas BMDMs were seeded at a density of 1 × 10^5^ in 12-well plates, respectively. After 24 hours of seeding, the culture medium was replaced with different formulations: control group (culture medium alone), lipopolysaccharide (LPS; Sigma-Aldrich, USA) + interferon-γ (IFN-γ; PeproTech, USA) group (culture medium with 1 µg/mL LPS and 30 ng/mL IFN-γ), and AnxA5-treated groups treatments with varying concentrations (0.5 µg/mL, 1 µg/mL, and 2 µg/mL) of AnxA5 along with LPS and IFN-γ for 48 hours (for Raw264.7 cells) and 6 hours (for BMDMs). Macrophage polarization was assessed using flow cytometry and quantitative Real-Time Polymerase Chain Reaction (qRT-PCR).

### Flow cytometry

Harvested cells were incubated with anti-mouse CD86 antibody (#159202, 1:40; BioLegend, USA) for 30 minutes at 4 °C. Data were collected using a BD Bioscience instrument and analyzed with CytExpert software (Beckman Coulter, USA).

### qRT-PCR

The mRNA levels of *Il1b*,* Il6*, Inducible nitric oxide synthase (*Inos*), *Tnfa*, arginase-1 (*Arg*) and *Tgfb* were quantified using the Total RNA Extraction Reagent (EZBioscience, USA). Initially, the reverse transcription master mix (EZBioscience, USA) was used to reverse-transcribe 1 μg of total RNA into cDNA. Subsequently, qRT-PCR was performed with the ROX2 plus master mix (EZBioscience, USA), with each sample replicated at least three times. The fold changes relative to *Gapdh* mRNA levels were calculated using the 2^-ΔΔCt^ method. The sequences of primers utilized in qRT-PCR are listed in Supplementary Table 1.

### Reactive oxygen species (ROS) measurement

ROS levels were accessed using the ROS assay kit (Beyotime, China) following the manufacturer's guidelines. Initially, cells were treated with 2,7-dichlorodihydrofluorescein diacetate (DCFH-DA) for 20 minutes at 37°C. Following three washes, cells were observed using a fluorescence microscope (Olympus Corporation, Japan). Subsequently, cells were detached and re-suspended for flow cytometry analysis (Beckman-Coulter, USA).

### Measurement of IFN-β concentration

BMDMs were treated as previously described, and the concentration of IFN-β in culture supernatants was measured with an enzyme-linked immunosorbent assay kit (#YZ-21263, Shanghai Yanzun Biotechnology, China).

### RNA sequencing (RNA-seq) for BMDMs

RNA-seq was conducted on three groups of BMDMs: Control, LPS+IFN-γ stimulated group (Model), and LPS+IFN-γ stimulated with AnxA5 treatment group (M+AnxA5) (Figure 3A). Total RNA was extracted and analyzed for fragments per kilobase per million mapped bases value by YOBIBIO (Shanghai, China). Differentially expressed genes (DEGs) were identified based on fold changes > 1.2 and adjusted *p <*0.05. Pathway analyses were conducted using the Kyoto Encyclopedia of Genes and Genomes (KEGG) and Gene Set Enrichment Analysis (GSEA) using the R software. The raw data has been deposited in the GEO database (GSE254402).

### Western blotting

Cellular proteins were isolated using RIPA supplemented with protease and phosphatase inhibitors (Beyotime, China). Subsequently, the proteins underwent separation via sodium dodecyl sulfate-polyacrylamide gel electrophoresis and were then transferred onto polyvinylidene fluoride membranes (Millipore, USA). Following incubation with primary and secondary antibodies conjugated with HRP (Abcam, UK), enhanced chemiluminescence (Pierce, USA) was used to visualize the membranes. The primary antibodies (Cell Signaling Technology, USA) used were anti-IκBα (#4812, 1:1000), anti-p-IκBα (#9246, 1:1000), anti-p65 (#8242, 1:1000), anti-p-p65 (#3033, 1:1000), anti-TLR4 (#14358, 1:5000) and anti-β-actin (#4967, 1:10000).

### AnxA5-FITC labeling

The Fluorescent Protein Labeling Kit (A10235, Invitrogen, Thermo Fisher Scientific, USA) was used to label FITC. Initially, the protein solution was combined with the reactive dye and agitated for 1 hour at room temperature. A 500 µL of 0.2 M, pH 9.4 bicarbonate buffer was passed through the column for buffer exchange. The protein mixture was then introduced into the prepared column for retrieval. The labeling efficiency was measured to be approximately 5.2, which was deemed satisfactory.

### AnxA5-FITC staining for BMDMs

To investigate the specific mechanism of AnxA5 inhibition on macrophage polarization towards the M1 phenotype, we utilized AnxA5-FITC to track AnxA5 locations during macrophage polarization. BMDMs were cultured with or without inflammatory factors (LPS+IFN-γ) and then treated with AnxA5-FITC for varying duration (10 minutes, 60 minutes, and 360 minutes). Following treatment, cells were fixed using 4% paraformaldehyde and observed with a fluorescence microscope (Leica SP8, Germany) and a Confocal microscope (Olympus IX73, Japan). These experiments were also conducted without calcium ions to assess their impact.

### LPS-FITC staining for BMDMs

To investigate the binding of LPS to macrophages following AnxA5 treatment, we cultured BMDMs in a standard growth medium containing inflammatory factors. They were then co-incubated with both inflammatory factors and AnxA5 for a duration of 6 hours. Subsequently, the cells were incubated with LPS-FITC (Sigma, USA) for 30 minutes, followed by DAPI staining (Southern Biotech, USA). The staining was observed with a fluorescence microscope (Leica SP8, Germany), and analysis was carried out utilizing flow cytometry (BD Bioscience, USA).

### Immunofluorescence staining

To investigate changes in cell surface TLR4 in macrophages before and after AnxA5 treatment with inflammatory factors, Raw264.7 cells were co-incubated with AnxA5-FITC and inflammatory factors. Furthermore, immunofluorescence staining of TGN46 or LAMP1 and TLR4 was conducted to access the dynamics of TLR4 transport in Raw264.7 cells. Following fixation and permeabilization, cells were stained with primary antibodies, including anti-TLR4 (#66350-1-Ig, 1:200, Proteintech, China), anti-TGN46 (#66477-1-Ig, 1:200, Proteintech, China) and anti-LAMP1 (#67300-1-Ig, 1:200, Proteintech, China) overnight at 4°C. Subsequently, a secondary antibody (1:200, Proteintech, China) was applied for 2 hours at room temperature. The staining was visualized using a Thunder imaging system (Leica, Germany).

### Dot-immunoblot studies

We conducted experiments using a dot-immunoblot method to investigate the direct interaction between AnxA5 and LPS or TLR4, following established protocols 15. The PVDF membrane was cut to an appropriate size, activated with methanol for 1 minute, immersed in ddH_2_O, and washed on a shaker for 15 minutes. Subsequently, the membrane was air-dried at room temperature until the water film disappeared. Experimental groups included the Blank group (PBS, 10 μL), negative control group (BSA, 2 μg/10 μL), positive control group (AnxA5, 2 μg/10 μL), and various concentrations of LPS groups (1 μg, 2 μg, 4 μg in 10 μL PBS). Each group of samples was added to the PVDF membrane and allowed to dry completely. The membrane was blocked with TBST containing 5% milk to prevent nonspecific binding, followed by incubation at 37°C for 2 hours. After washing the PVDF membrane 5 times with TBST, each time for 5 minutes, the membrane was incubated with AnxA5 (2 μg/mL) overnight at 4°C and rewashed. Subsequent steps were conducted as Western blot with the primary anti-AnxA5 antibody (#11060-1-AP, 1:5000, Proteintech, China). The experiment involving TLR4 followed a similar protocol, with TLR4 protein (Thermo Fisher Scientific, USA) at concentrations of 1 μg, 2 μg, and 4 μg in 10 μL PBS.

### Animal study

Male Sprague-Dawley (SD) rats (7-8 weeks; 332.67 ± 5.02 g) were procured from Vital River Laboratory Animal Technology Co., Ltd (SYXK-2022-0020). All rats had no restrictions on food or water access (temperature: 20-25°C, humidity: 40-70%, light: 12 hours/day). The Animal Care and Experiment Committee of Shanghai Ninth People's Hospital approved the experiments. The rats were randomly divided into four groups (n = 6 per group): control, model, AnxA5^lowdose^, and AnxA5^highdose^. The control group remained untreated from Day -7 to Day 0. Rats in other groups were induced with OA by intra-articular administration of 50 µL monosodium iodoacetate (MIA, Sigma, USA) solution at a concentration of 40 mg/mL. Subsequently, the rats received four intra-articularly injected of either saline, AnxA5^lowdose^ (1 mg/mL) or AnxA5^highdose^ (3 mg/mL), each administered in a volume of 50 µL. Pressure pain tests were conducted on Days 7, 21, 35, and 49, and all surviving animals were euthanized on Day 54.

### Visual gait analysis

Gait analysis was conducted using a VisuGait system (Xinruan XR-FP101, Shanghai) 16, 17. Rats' movements were recorded by video cameras positioned below the animals at 120 frames per second while traversing an illuminated glass platform enclosed by dark plastic walls. The analysis did not include trials in which the rats exhibited uneven pacing or changed direction. Footprints were categorized and measured by trained technical personnel unaware of the rat treatments, utilizing the VisuGait analysis software (Version 2.0).

### Histological analyses

The left knee joint of rats was fixed in 4% paraformaldehyde at 4°C for 24 hours and decalcified in 10% EDTA for 4 weeks. Sagittal sections (5 µm) were obtained after paraffin embedding. Hematoxylin-eosin (HE) and Safranin O-fast green (S&F) staining were used to observe structure. The Osteoarthritis Research Society International (OARSI) evaluated the S&F structural scoring based on joint pathology scoring standards 18. The HE structural score was assigned on a scale of 1 to 4 (1 = slight; 2 = mild; 3 = moderate; 4 = severe) by randomly selecting visual fields. Glycosaminoglycan (GAG) was observed by staining sections with toluidine blue (Solarbio, China).

Sections were subjected to antigen retrieval using the citric acid buffer (pH = 6.0, Beyotime, China), followed by permeabilization with 0.2% Triton (Triton X-100, Solarbio, China) and blocking of peroxidase with 3% H_2_O_2_. Following blocking with goat serum (Solarbio, China), tissue sections were incubated with antibodies targeting CD86 (#sc-28347, 1:100, Santa Cruz, USA) and CD206 (#24595, 1:200, Cell Signaling Technology, USA) overnight. Subsequently, sections were incubated with HRP-labeled secondary antibody (Beyotime, China) for 30 minutes at 37°C. Visualization was then done using 3, 3'-Diaminobenzidine (DAB Substrate Kit, Burlingame, USA), followed by counterstaining with hematoxylin. Each section was imaged in three randomly using light microscopy (Nikon Eclipse 90i, Japan). The number of CD86^+^ and CD206^+^ cells was analyzed with Image-Pro Plus (Rockville, USA).

### Isolation and culture of mouse primary chondrocyte

Primary chondrocytes were obtained and confirmed as described previously 7. Chondrocytes were cultured in F-12/DMEM (Invitrogen, Thermo Fisher Scientific, USA) and subjected to an inflammatory environment by adding 10 nM IL-1 and 10 nM TNF-α (PeproTech, USA).

### Chondrocyte YO-PRO-1 staining

The YO-PRO-1 probe (Beyotime, China) was utilized to evaluate apoptosis and necrosis in chondrocytes. In summary, chondrocytes were immersed in Yo-PRO-1 detection working solution and incubated at 37°C for 20 minutes. Subsequently, the cells were examined using flow cytometry (EX/Em = 491/509 nm).

### Terminal deoxynucleotidyl transferase-mediated dUTP Nick-End Labeling (TUNEL) assay

Chondrocytes were first fixed with 4% paraformaldehyde and then permeabilized with 0.3% Triton X-100. Subsequently, the cells were incubated with the TUNEL detection solution (Beyotime, China) at 37°C for 60 minutes, and the nuclei were counterstained with DAPI (Southern Biotech, USA).

### Calcein-acetoxymethyl (AM)/Propidium Iodide (PI) assay

The Calcein-AM/PI assay was conducted to evaluate chondrocyte viability and identify cell death using the Calcein AM/PI Cell Viability assay kit (Beyotime, China). Briefly, chondrocytes were exposed to the Calcein AM/PI detection solution and observed with the fluorescence microscope (Leica SP8, Germany). Image-Pro Plus was used to analyze the average fluorescence intensity in five fields.

### Statistical analysis

Two groups were compared using paired or unpaired Student's t-tests (parametric or non-parametric tests). One-way analysis of variance (ANOVA, a parametric test) was utilized for three or more groups with parametric data. In contrast, non-parametric data (OARSI and synovium scores) were analyzed using the Kruskal-Wallis test. As shown in Figure 1, Dunnett's test was conducted for post hoc comparisons, with the LPS+IFN-γ group set as the control group. In other experiments, Tukey's test was employed for post hoc comparisons. All graphs were generated using Prism 9 (GraphPad Software, USA). The results are presented as the mean ± standard deviation (SD), with *p<*0.033 considered statistically significant (^*^p<0.033, ^**^p<0.002, ^***^p<0.001) and ns indicating no significance. The statistical differences between the control and model groups, as well as between the model group and each treatment group, were labeled in the figures. Treatment groups without significant statistical differences were not labeled on the figures.

## Results

### AnxA5 as a potential regulator of macrophage polarization in CEFFE

CEFFE inhibited the M1 polarization of Raw264.7 cells induced by LPS and IFN-γ *in vitro*, although the specific mechanism remains unclear. CEFFE consists of over 1700 soluble proteins, suggesting the involvement of one or more soluble inhibitory factors in the observed phenotype changes. To identify these proteins that regulate macrophage polarization, we purified the proteins using three-step anion exchange and cation exchange liquid chromatography. The expression of M1 representative genes *Il1b* and *Inos* in Raw264.7 cells were detected using qRT-PCR after stimulation with LPS and IFN-γ (Figure 1A). Following the third round of purification, the 3-1-3 fraction demonstrated a strong inhibitory effect on inflammatory factors in M1 macrophages (Figure 1B, C, and D). Proteins in the 3-1-3 fraction were identified using LC-MS/MS, and AnxA5 was identified among the analyzed proteins (Table 1). Recent studies have suggested that AnxA5 positively affects inflammatory diseases such as inflammatory bowel disease and non-alcoholic liver fibrosis and may regulate macrophage polarization.

### AnxA5 mainly regulates the polarization of the macrophage M1 phenotype

**Figure 2:**
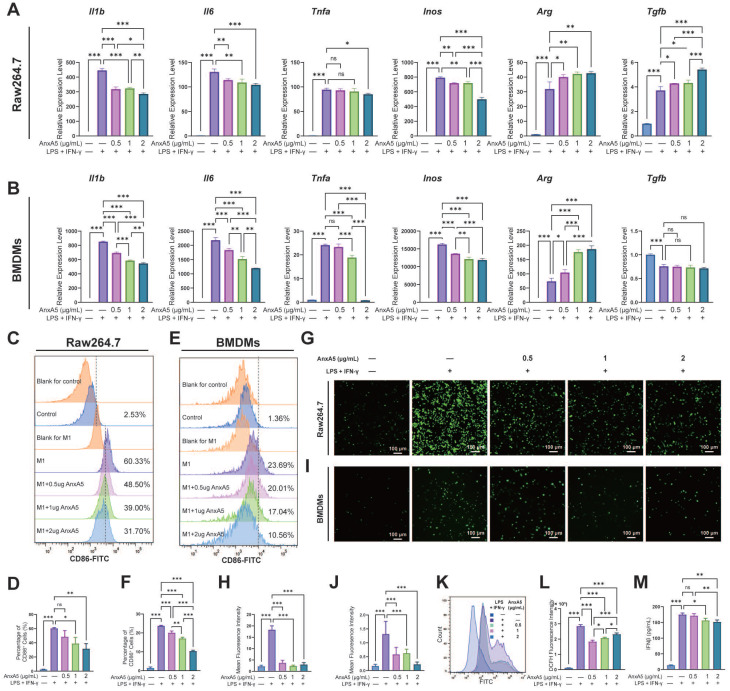
AnxA5 Suppresses M1 Macrophage Polarization. **A**, **B**. Raw264.7 cells and BMDMs were exposed to 1 μg/mL LPS and 30 ng/mL IFN-γ, then treated with different concentrations of AnxA5 (0.5, 1, 2 μg/mL) for 48 hours and 6 hours, respectively. The mRNA levels of *Il1b*, *Il6*, *Tnfa*, *Inos*, *Arg* and *Tgfb* were determined by qRT-PCR, showing a decrease in the expression of M1-associated inflammatory mediator genes in both cell types. **C**, **D**. Analysis of CD86 flow cytometry data revealed a decrease in the percentage of M1-like cells in the Raw264.7 cell model following AnxA5 treatment. The numbers on the graph represented the mean from three experiments. **E**,** F**. Analysis of CD86 flow cytometry data revealed a decrease in the percentage of M1-like cells in the BMDMs cell model following AnxA5 treatment. The numbers on the graph represented the mean from three experiments. **G**, **H**. Representative images of Raw264.7 cells stained with DCFH-DA, and the mean fluorescence intensity was quantified. **I**, **J**. Representative images of BMDMs stained with DCFH-DA were analyzed for mean fluorescence intensity. **K**,** L**. Flow cytometry results and quantification of mean fluorescence intensity in Raw264.7 cells. **M**. IFN-β levels in the supernatant of BMDMs cell culture were measured using ELISA. Scale bars were noted on the right bottom corner of each image. Data presented as mean ± standard deviation (n=3 per group). ^*^p < 0.033; ^**^p < 0.002; ^***^p < 0.001; ns, not significant.

To assess the impact of AnxA5 on macrophage polarization, Raw264.7 cells and BMDMs were treated with LPS and IFN-γ along with 0.5 µg/mL, 1 µg/mL, and 2 µg/mL of AnxA5. Analysis through qRT-PCR revealed that the expression of pro-inflammatory cytokines, including *Il1b, Il6, Inos,* and* Tnfa,* was significantly higher in the LPS+IFN-γ group than in the control group (Figure 2A, B). However, this expression dose-dependently decreased with AnxA5 co-incubation. Conversely, there was an increase in the expression of anti-inflammatory cytokines, including *Arg* and *Tgfb,* in the LPS+IFN-γ+AnxA5 group (Figure 2A, B). Flow cytometry results were consistent with the qRT-PCR results, showing that LPS+IFN-γ significantly induced M0 macrophages to polarize towards the M1 phenotype (CD86^+^, pro-inflammatory cells), with a substantial increase in percentage from 2.53 ± 0.26% to 60.33 ± 1.12% in Raw264.7 and from 1.36 ± 0.63% to 23.69 ± 0.38% in BMDMs (Figure 2C-F). However, a decrease in the proportion of M1 macrophages was noted in Raw264.7 cells from 60.33 ± 1.12% to 48.50 ± 8.45%, 39.00 ± 8.64%, and 31.70 ± 7.12%, and in BMDMs from 23.69 ± 0.38% to 20.01 ±1.27%, 17.04 ± 0.66%, and 10.56 ± 0.44% with increasing concentrations of AnxA5 (0.5, 1 and 2 µg/mL), indicating a dose-dependent relationship between AnxA5 concentration and macrophage phenotype reduction (Figure 2C-F). A decrease in intracellular ROS levels in Raw264.7 cells and BMDMs after co-incubation with AnxA5 was observed using fluorescence microscopy and flow cytometry (Figure 2G-L). Additionally, AnxA5 was found to lower the concentration of IFN-β in the cell supernatant (Figure 2M). These findings indicated that AnxA5 regulated macrophage polarization by inhibiting M1 macrophage development.

### AnxA5 inhibits M1 polarization by suppressing toll-like receptor (TLR) signaling

**Figure 3:**
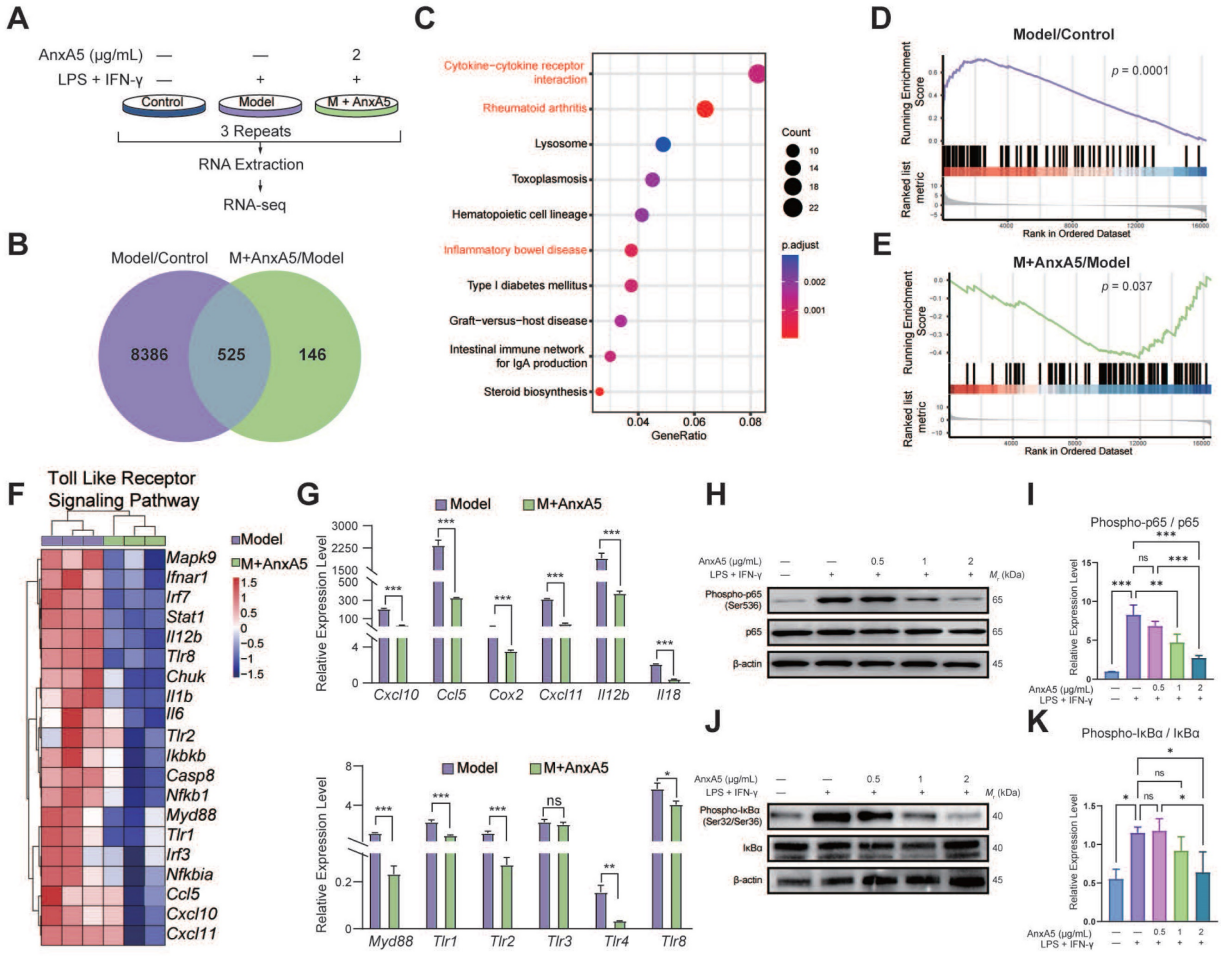
AnxA5 Inhibits TLR Signaling Pathway and Downstream NF-κB in Macrophage. **A**. Schematic representation of RNA-sequence analysis. **B**. Venn diagram. **C**. Analysis of 525 DEGs using the KEGG. **D, E**. GSEA analysis. **F**. DEGs involved in TLR pathway. **G**. Validation of DEGs using qRT-PCR. **H-K**. Western blot analysis showing levels of P-p65, p65, P-IκBα, IκBα, and β-actin after stimulation with LPS+IFN-γ with or without AnxA5 in BMDMs. Data presented as mean ± standard deviation (n=3 per group). ^*^p < 0.033; ^**^p < 0.002; ^***^p < 0.001; ns, not significant.

Differential gene expression analysis identified 8,911 DEGs between the Control and Model groups and 671 DEGs between the Model and M+AnxA5 groups, with 525 shared between them (Figure 3B). Pathway enrichment analysis indicated a significant enrichment of shared DEGs related to inflammation-related diseases such as rheumatoid arthritis and inflammatory bowel disease (Figure 3C). GSEA revealed significant activation of the TLR signaling pathway in the Model group, which was notably suppressed by AnxA5 treatment (Figure 3D, E). AnxA5 treatment resulted in a significant downregulation of genes like *Tlr4*,* Myd88*,* Ccl5, and Cxcl12* compared to those in the model group (Figure 3F, G). The TLR4 signaling pathway is crucial for LPS-induced macrophage polarization, mediated by MyD88-dependent and MyD88-independent pathways involving MyD88 and TRIF, respectively. Activation of TLR4 triggers NF-κB activation and the release of pro-inflammatory cytokines. AnxA5 treatment effectively mitigated NF-κB activation through the MyD88-dependent pathway (Figure 3H-K). These findings suggest that AnxA5 inhibits M1 macrophage polarization by modulating the TLR pathway and downstream signaling, possibly through direct or indirect interactions with LPS or TLR4.

### AnxA5 inhibits macrophage activation by promoting TLR4 internalization and lysosomal degradation

**Figure 4:**
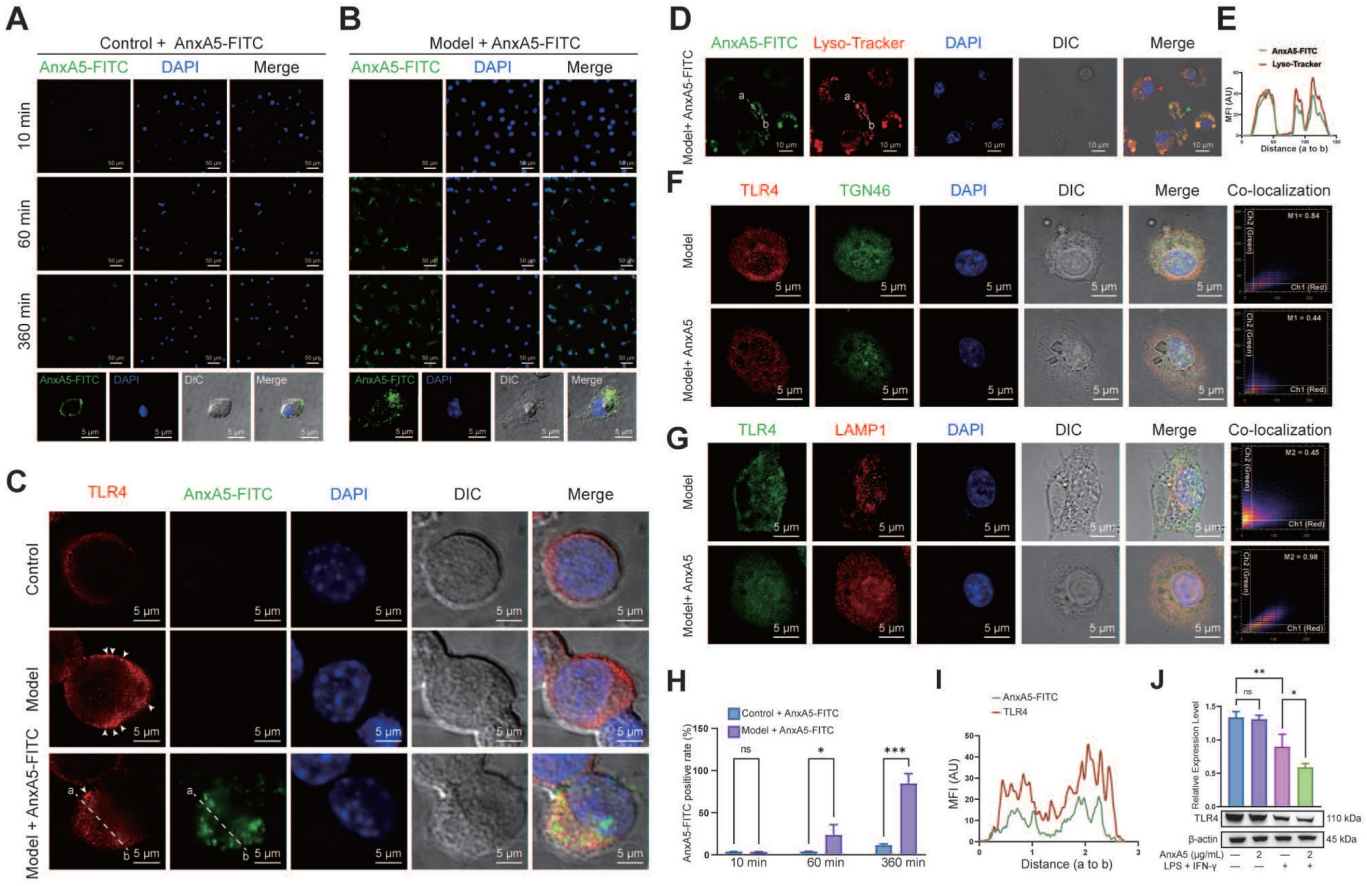
AnxA5 Facilitates TLR4 Internalization and Lysosomal Degradation. **A**, **B**, and **H**. Representative images of 2 μg/mL AnxA5-FITC staining at 10 minutes, 60 minutes, and 360 minutes in the presence and absence of inflammatory factors. Immunofluorescence images represent five different fields of views. **C** and **I**. Representative immunofluorescence images and co-localization analysis of TLR4 following a 6-hour treatment with 2 μg/mL AnxA5-FITC and inflammatory factors. Nuclei were stained with DAPI (blue) and white arrowheads indicated TLR4 location on the cell membrane. Line graphs represent mean fluorescence intensity (MFI), depicting distance from 'a' to 'b'. Immunofluorescence images represent five different fields of views. **D**. Representative field fluorescence images of Lyso-tracker staining after a 6-hour treatment with 2 μg/mL AnxA5-FITC and inflammatory factors. Nuclei were stained with DAPI (blue). Red arrows indicated cells with no internalized AnxA5, green arrows indicated AnxA5 bound to cell membrane, and yellow arrows indicated internalized AnxA5. **E**. Co-localization analysis of AnxA5-FITC and Lyso-tracker. Line graph represented MFI, depicting distance from 'a' to 'b'. **F, G.** Representative images of TGN46 and LAMP1 immunofluorescence staining after 6-hour treatment with 2 μg/mL AnxA5 and inflammatory factors. Immunofluorescence images represent five different fields of views. **J.** Western blot results of TLR4 in macrophage after AnxA5 treatment. Scale bars were noted on the right bottom corner of each picture. Data presented as mean ± standard deviation (n=3 per group). ^*^p < 0.033; ^**^p < 0.002; ^***^p < 0.001; ns, not significant.

To explore the potential direct or indirect relationship between AnxA5 and LPS or TLR4, we conducted AnxA5-FITC staining experiments to examine its localization in macrophages, both with or without inflammatory factors. The results showed that in the absence of inflammatory factors, AnxA5 had minimal cell binding and was mainly located on the cell surface (Figure 4A). However, in the presence of inflammatory factors, AnxA5 gradually internalized from the cell surface, with an internalization efficiency reaching 85.54 ± 11.6% over time (Figure 4B, H).

Based on these observations, we postulated that AnxA5 influences macrophage polarization by directly interacting with LPS or TLR4. Our experimental data demonstrated that AnxA5 reduced LPS-FITC binding to the cell surface (Supplementary Figure 2A, B). Furthermore, a direct interaction between AnxA5 and LPS was observed, suggesting that AnxA5 may attenuate the activity of LPS through this interaction (Supplementary Figure 2C), which is consistent with previous experimental findings 15. We further investigated the effect of AnxA5 on the sub-cellular localization and expression of TLR4. In the presence of inflammatory factors, cells treated with AnxA5-FITC displayed increased internalization of TLR4, and AnxA5-FITC co-localized with TLR4 (Figure 4C, I). These findings suggest that AnxA5 internalization promotes TLR4 internalization, reducing signal transduction via the MyD88-dependent pathway. It is essential to note that while LPS can activate downstream MyD88-dependent signaling from cell surface TLR4, leading to the production of pro-inflammatory cytokines (e.g., *Il1*, *Il6*, and *Tnfa*), it can also activate MyD88-independent and TRIF-dependent activation of IRFs, regulating IFN-inducible genes (e.g., *Cxcl10* and *Ifnb*) through TLR4 internalization.

Our study indicated that internalized AnxA5 was predominantly co-localized with cellular lysosomes, suggesting its potential involvement in facilitating TLR4 binding to the cell surface and subsequent entry into lysosomes (Figure 4D, E). Before LPS treatment, TLR4 was primarily localized to the cell membrane; however, upon LPS stimulation, TLR4 was internalized and exhibited a dynamic transport process. During the early stages of LPS treatment, TLR4 remained abundant in the retrograde Golgi network pathway (TGN46-positive) (Figure 4F). In AnxA5-treated macrophages, internally localized TLR4 co-localized with lysosomes positive for LAMP1, implying that AnxA5 may facilitate the lysosomal degradation of TLR4 (Figure 4G). Additionally, these cells' overall expression of TLR4 decreased (Figure 4J). However, the experimental results indicated the absence of a significant direct interaction between AnxA5 and TLR4 (Supplementary Figure 2D). Moreover, the inhibition of macrophage polarization was calcium-dependent, as the inhibitory effect of AnxA5 on polarization was abolished when the cells were cultured in a calcium-free medium (Supplementary Figure 2E).

### Treatment effect of AnxA5 on MIA-induced OA rats

**Figure 5:**
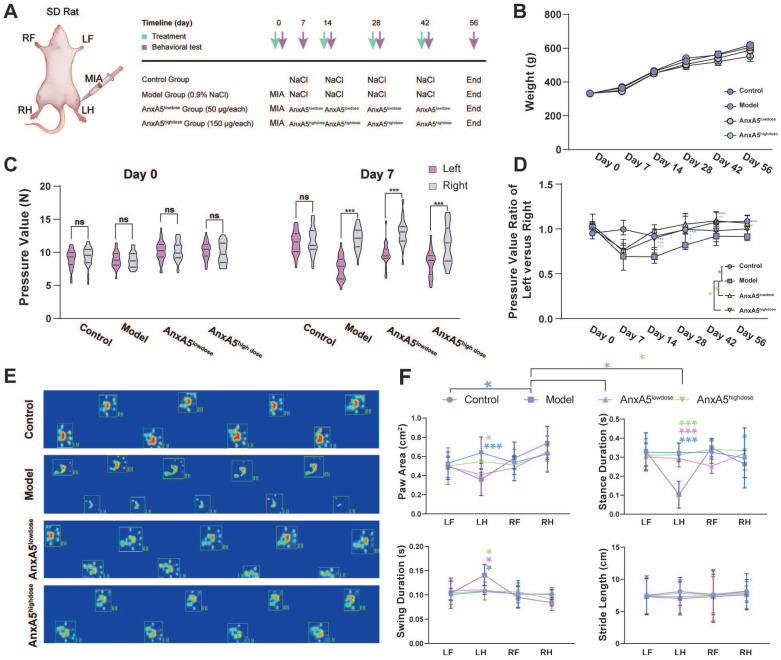
AnxA5 Therapy for MIA-Induced OA Rat Symptoms. **A.** Schematic of MIA intra-articular injection. Limb labels: Right Front (RF), Left Front (LF), Right Hind (RH), Left Hind (LH). **B.** No significant body weight changes observed among groups at different time points. **C.** Claw retraction pressure changes post-MIA injection. **D.** Quantitative analysis of pressure value ratio between left and right limbs over time across groups. **E.** Representative gait images of rat hind limbs in different groups.** F.** Analysis of paw area, stance duration, swing duration and stride length for RF, RH, LF, and LH in different groups. Data presented as mean ± standard deviation (n=6 per group). ^*^p < 0.033; ^**^p < 0.002; ^***^p < 0.001; ns, not significant.

We investigated the effects of AnxA5 treatment using a rat model of OA induced by intra-articular administration of MIA and performed behavioural and histological evaluations (Figure 5A). The results showed that neither MIA injection nor AnxA5 treatment affected the body weight of rats throughout the entire experiment (Figure 5B). The success of the rat OA model was verified using a pressure pain test on Day 7, which demonstrated a significant decrease in the pressure threshold of the left knee joint (Figure 5C). Throughout the experiment, the model group exhibited a gradual recovery of pressure pain in the left leg, yet the pain pressure threshold remained lower than that of the control group. Low-dose AnxA5 improved OA pain symptoms throughout the experiment, whereas high-dose AnxA5 showed pain relief effects in the left leg in the early stages (Day 7 to Day 28) (Figure 5D). Catwalk gait analysis (Figure 5E and Supplementary Figure 3) on Day 56 confirmed pain relief in the rat OA model after AnxA5 treatment, as evidenced by decreased pressure on the left leg as demonstrated by thermal imaging and gait patterns. Additional measurements showed significant decreases in paw area and stance duration, alongside significant increases in swing duration for the OA rats compared to those in the control rats, with significant improvements observed in both low- and high-dose AnxA5 groups (Figure 5F). These findings demonstrate that AnxA5 treatment effectively reduces pain symptoms induced by the intra-articular injection of MIA in the knee joint.

**Figure 6:**
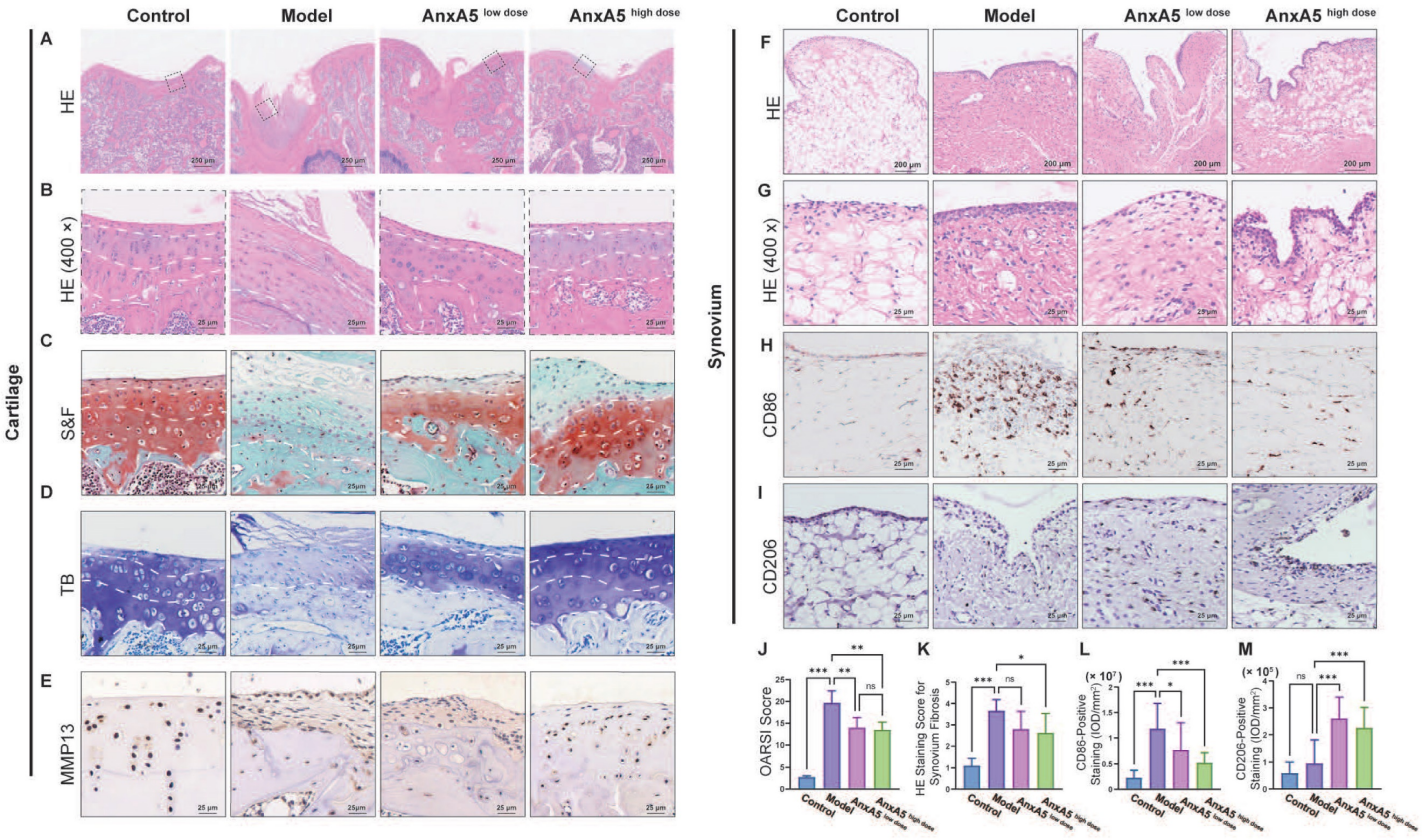
Evaluation of cartilage and synovium. **A, B** HE staining. **C, J** S&F staining and quantitative analysis based OARSI standard. **D** TB staining. **E** Anti-MMP13 staining. **F, G and K** HE staining for synovial tissue and quantitative analysis of synovium fibrosis. **H, L** Anti-CD86 staining and quantitative analysis of CD86^+^ ratio of synovium. **I, M** Anti-CD206 staining and quantitative analysis of CD206^+^ ratio of synovium. Scale bars were noted on the right bottom corner of each picture. Data presented as mean ± standard deviation (n=6 per group). ^*^p < 0.033;^ **^p < 0.002; ^***^p < 0.001; ns, not significant.

To study the therapeutic mechanisms of AnxA5, we performed histological analyses of the synovium and cartilage sections. AnxA5 treatment notably restored the overall morphology of these sections closer to that of the control group, as observed by HE and S&F staining (Figure 6A, B, and C). In the model group, the internal structure of the articular cartilage was altered and replaced with fibrous tissue. In the AnxA5 treatment group, the progression of cartilage degeneration was ameliorated, as shown by the OARSI scoring and toluidine blue staining (Figure 6D, J). Moreover, MMP13 immunohistochemistry revealed a significant decrease in MMP13 expression in the cartilage of the AnxA5 treatment group compared to the model group, indicating the protective effect of AnxA5 on the cartilage (Figure 6E).

Furthermore, AnxA5 treatment reduced the rate of fibrosis in the synovial tissue, as shown by HE staining (Figure 6F, G, and K), significantly decreased the infiltration of M1 macrophages (CD86^+^ cells; Figure 6H and L), and increased the proportion of M2 macrophages (CD206^+^ cells; Figure 6I and M), as shown by CD86 and CD206 staining, respectively. These results suggest that AnxA5 positively affects the cartilage matrix, synovial macrophages, and fibroblasts.

### AnxA5 exhibits protective effects against chondrocyte necrosis and apoptosis

**Figure 7:**
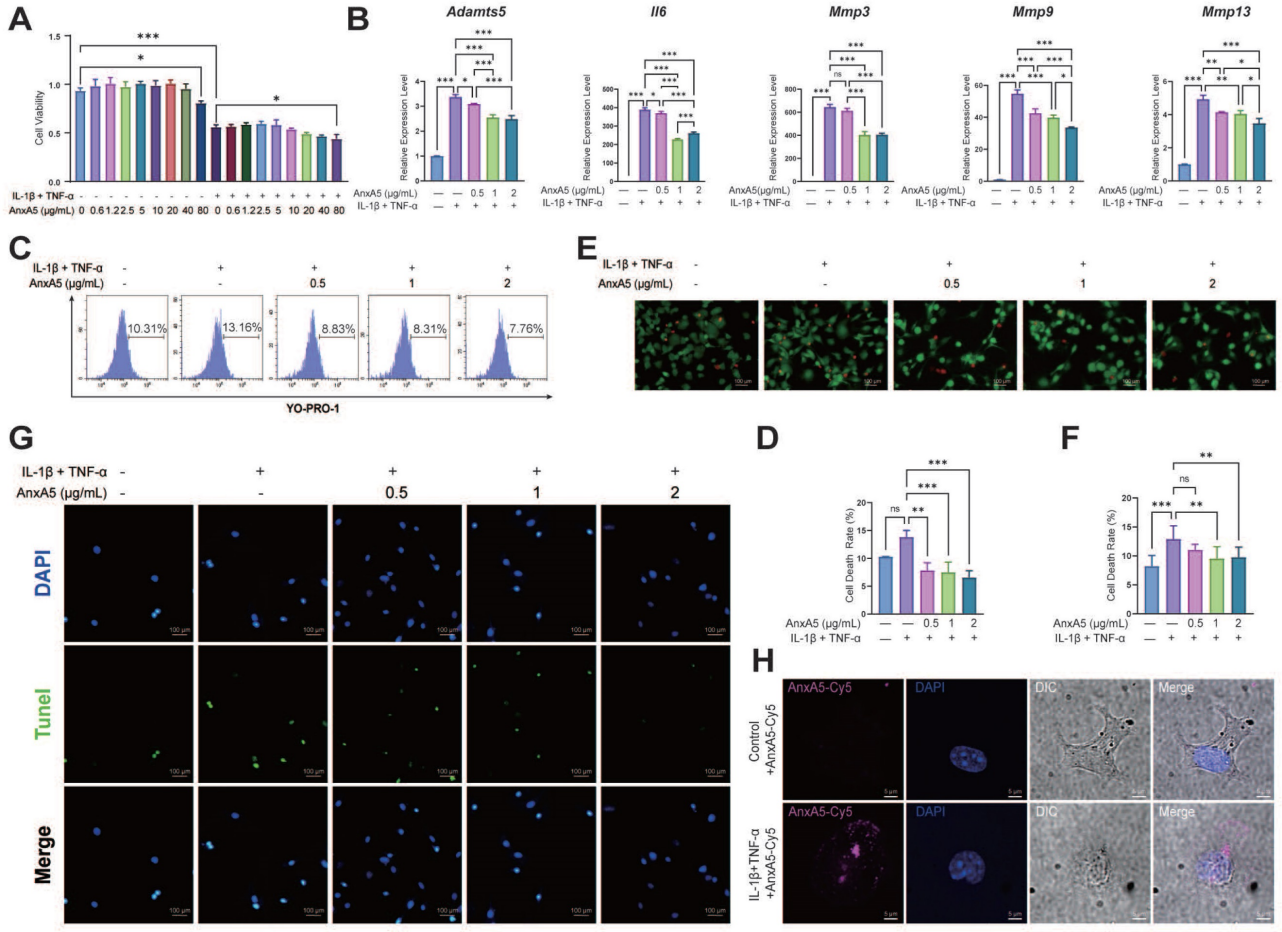
The protective Effects of AnxA5 in Chondrocytes. **A** AnxA5 at low-to-moderate doses did not exhibit cytotoxicity in mouse chondrocytes. **B** AnxA5 dose-dependently reduced mRNA levels of *Adamts5, IL6, Mmp3, Mmp9,* and* Mmp13* induced by inflammatory factors, as analyzed by qRT-PCR. **C, D** AnxA5 decreased necrosis and apoptosis rates in chondrocytes under inflammatory conditions as analyzed by YO-PRO staining. **E, F** AnxA5 reduced necrosis rates in chondrocytes under inflammatory conditions assayed by AM/PI staining. **G** AnxA5 reduced apoptosis rates in chondrocytes under inflammatory conditions assayed by TUNEL staining. **H** Representative images of 2 μg/mL AnxA5- Cy5 staining at 48 hours in the presence and absence of inflammatory factors. Scale bars were noted on the right bottom corner of each picture. Pictures of immunofluorescence are the representatives of five different visual fields**.** ^*^p < 0.033; ^**^p < 0.002;^ ***^p < 0.001; ns, not significant.

The CCK-8 assay showed that AnxA5 had no significant effect on chondrocyte proliferation in either inflammatory or non-inflammatory environments (Figure 7A). However, AnxA5 did inhibit inflammation and matrix metalloproteinases in an inflammatory environment (Figure 7B), which is consistent with observations from animal experiments. Moreover, AnxA5 moderately reduced the levels of chondrocyte necrosis and apoptosis caused by inflammatory factors (Figure 7C-G). Under normal conditions, AnxA5-Cy5 barely entered the chondrocytes. However, in an inflammatory environment, some AnxA5-Cy5 was distributed on the cell surface, and some entered the cells (Figure 7H). These results suggest a protective role for AnxA5-Cy5 in chondrocyte necrosis and apoptosis, which is potentially linked to its interaction with membranes rich in phosphatidylserine (PS).

## Discussion

In this study, we demonstrated for the first time that AnxA5, a calcium-dependent phospholipid-binding protein, is a critical protein in CEFFE that effectively alleviates synovial inflammation and cartilage degradation in a rat model of OA. *In vitro* experiments revealed that AnxA5 may promote the internalization of TLR4 in the presence of calcium ions (Figure 4). This binding of AnxA5 to TLR4 leads to the lysosomal degradation of TLR4, reducing the overall TLR4 expression level in the cells 19-21. As a result, the sustained survival of M1 macrophages is diminished, leading to the suppression of downstream inflammatory mediator expression and ROS release 22-24. Furthermore, AnxA5 exhibits protective effects against chondrocyte necrosis and apoptosis, indicating its potential role in preserving cartilage survival 25.

Previous studies have reported that AnxA5 can bind to PS-rich membranes, leading to high concentrations of calcium ions on the membrane and subsequent changes in lipid dynamics and organization, eventually causing a membrane phase transition 26. However, the precise mechanism by which AnxA5 inhibits M1 macrophage polarization remains unclear 27. *In vitro* experiments suggested that AnxA5 was an effective negative regulator of the MyD88- and TRIF-dependent TLR4 signaling pathways (Figure 3). TLR4 is mainly localized on the plasma membrane before LPS treatment and then internalized and exists in early endosomes positive for EEA1, the trans-Golgi network positive for TGN46, and late endosomes/lysosomes positive for LAMP1, showing dynamic transport 28-30. AnxA5, with its inherent structural curvature in the presence of calcium ions, may facilitate cellular membrane endocytosis 31, 32, 14, 33. By coupling to TLR4 receptors on the cell membrane, AnxA5 reduces the recycling of TLR4 to the trans-Golgi network and promotes its degradation in lysosomes. This evidence aligns with previous findings showing that AnxA5 can target HUVEC exposure to PS, thereby inducing TLR4 internalization and inhibiting inflammation 19. However, since AnxA5 does not exhibit protein specificity for TLR4 but couples through membrane structures, we predicted that AnxA5 might also regulate other TLR or membrane receptor signaling pathways involving endosome-lysosome transport. However, the detailed mechanism underlying AnxA5-mediated membrane transport requires further investigation.

The structural characteristics of AnxA5 may be related to its protective effects 13, 32. AnxA5 may self-assemble into lattices and further stabilize the membrane into a gel phase, facilitating membrane repair and maintaining membrane integrity 13, 14. In a non-inflammatory environment, AnxA5 does not affect the survival or proliferation of the chondrocytes. In inflammatory conditions, AnxA5 has decreased cell death and apoptosis rates while safeguarding chondrocytes from metabolic imbalance (Figure 6E, Figure 7). It is hypothesized that, in the presence of inflammation, the integrity of the cell membrane is compromised, leading to increased cell death. AnxA5 may attach to PS on the cell membrane, forming a lattice network structure that hinders further damage 34, 35. This action helps preserve membrane integrity and protects chondrocytes 31, 36. Nevertheless, previous studies have suggested an association between the overexpression of AnxA5 and the promotion of apoptosis in chondrocytes, implying potential differences in the mechanisms between exogenous addition and AnxA5 37, 38.

CEFFE, as a mixture library, has shown effectiveness in various disease models, including orthopedic diseases such as OA and osteoporosis, dermatological diseases such as wound healing and photo-aging, and gynecological diseases such as premature ovarian failure 39-42, 8. Chronic inflammation is a common and significant pathological condition associated with these diseases. Identifying AnxA5 as an active protein in CEFFE that inhibits M1 macrophage polarization may help inform its potential therapeutic applications in these diseases (Figure 1, Figure 2). In the OA rat model, CEFFE and AnxA5 injections effectively relieved pain symptoms (Figure 6). AnxA5 injection demonstrated effects comparable to CEFFE in regulating macrophages, inhibiting M1 polarization, and promoting M2 polarization. However, AnxA5 injection showed inferior efficacy in cartilage regeneration indicators, such as type II collagen, compared with CEFFE injection. This suggests that the potential additional components in CEFFE contribute to its effects, which requires further experimental confirmation. Therefore, other biological factors associated with CEFFE should be investigated in future studies. For instance, if the anti-apoptotic and pro-chondrogenic growth factors in CEFFE could be identified, a precise combination of protein mixtures with anti-inflammatory, anti-apoptotic, and pro-chondrogenic activities could achieve better therapeutic outcomes in OA than using AnxA5 alone. The same approach could be used to develop new drugs for other diseases.

In conclusion, this study highlights the potential therapeutic role of AnxA5 in macrophage polarization and offers new insights into its mechanism of action. The protective effects of AnxA5 against chondrocyte necrosis and apoptosis add to its potential as a therapeutic agent for preserving cartilage health. These findings pave the way for further investigation into the development of AnxA5-based treatments for inflammatory and degenerative diseases. Additional studies are required to elucidate the precise molecular mechanisms and potential clinical applications of AnxA5.

## Supplementary Material

Supplementary figures and table.

## Figures and Tables

**Table 1:** List of Identified Proteins (MASCOT Score > 20)

Identified protein	Theoretical M (kDa)	Score	Peptide matched
Hornerin	282.390	68.022	3
Annexin A5	35.936	58.412	5
Desmoglein-1	113.750	26.999	2
Dermcidin	11.284	25.296	2
DNA polymerase	21.816	24.5	1

## Abbreviations

AnxA5: Annexin A5
α-MEM: α-Minimum Essential Medium
BMDMs: Bone marrow derived macrophages
CEFFE: Cell-free fat extract
Calcein-AM/PI: Calcein-acetoxymethyl/Propidium iodide
DAMPS: Damage-associated molecular patterns
DMEM: Dulbecco's Modified Eagle Medium
FBS: Fetal bovine serum
GAG: Glycosaminoglycan
HE: Hematoxylin-eosin
MIA: Monosodium iodoacetate
MFI: Mean fluorescence intensity
ANOVA: One-way analysis of variance
OA: Osteoarthritis
OARSI: Osteoarthritis Research Society International
PS: Phosphatidylserine
qRT-PCR: Quantitative real-time polymerase chain reaction
ROS: Reactive oxygen species
RNA-seq: RNA sequencing
S&F: Safranin O-fast green
SD rats: Sprague-Dawley rats
SD: Standard deviation
TLRs: Toll-like receptors
TUNEL: Transferase-mediated dUTP nick-end labeling
IFN: Type I interferon
