# Supplementary material for: Annexin A5 Derived from Cell-free Fat Extract Attenuates Osteoarthritis via Macrophage Regulation
Source: Int J Biol Sci. 2024 May 19;20(8):2994–3007. doi: 10.7150/ijbs.92802 (PMC11186356; doi:10.7150/ijbs.92802)
Supplement: Supplementary file 1 — Supplementary figures and table. [file ijbsv20p2994s1.pdf]

Supplementary Information

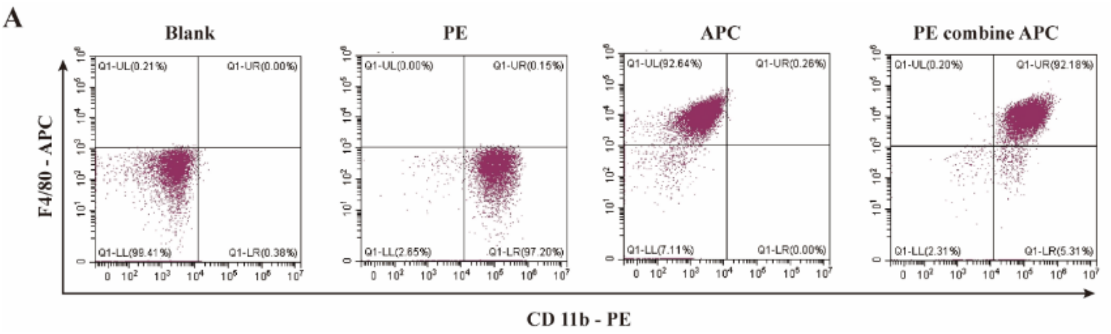

Supplementary Figure 1. Identification of BMDMs using flow cytometry.

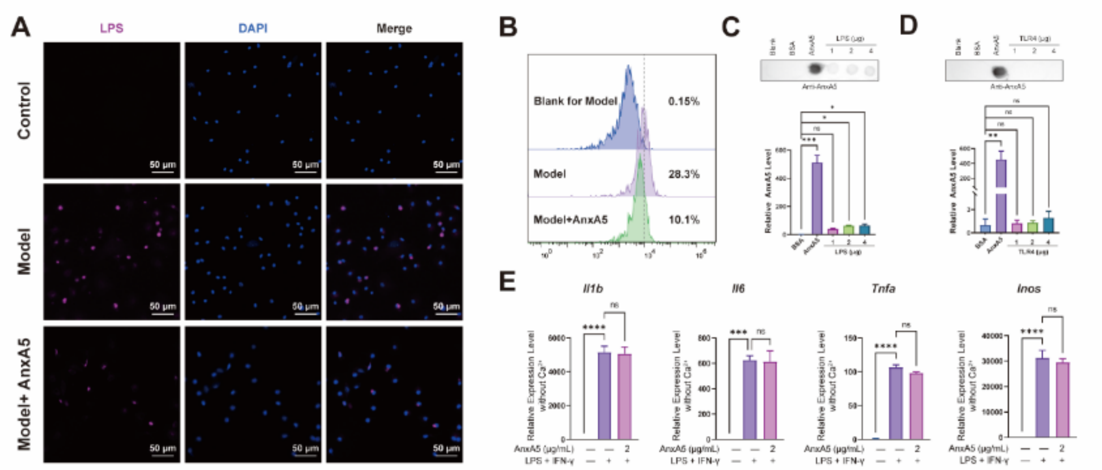

Supplementary Figure 2. AnxA5 Activity Requires Calcium Ions. **A, B** Representative fluorescence microscopy images and flow cytometry analysis of LPS-FITC staining in AnxA5-treated macrophages. **C** Dot-immunoblot between AnxA5 and LPS. **D** Dot-immunoblot between AnxA5 and TLR4. **E** The ability of AnxA5 to inhibit M1 macrophage polarization was lost in calcium ion-deficient culture medium. Scale bars were noted on the right bottom corner of each picture. Data presented as mean ± SD (n=3 per group). $^{*}p < 0.033$; $^{**}p < 0.002$; $^{***}p < 0.001$; ns, not significant.

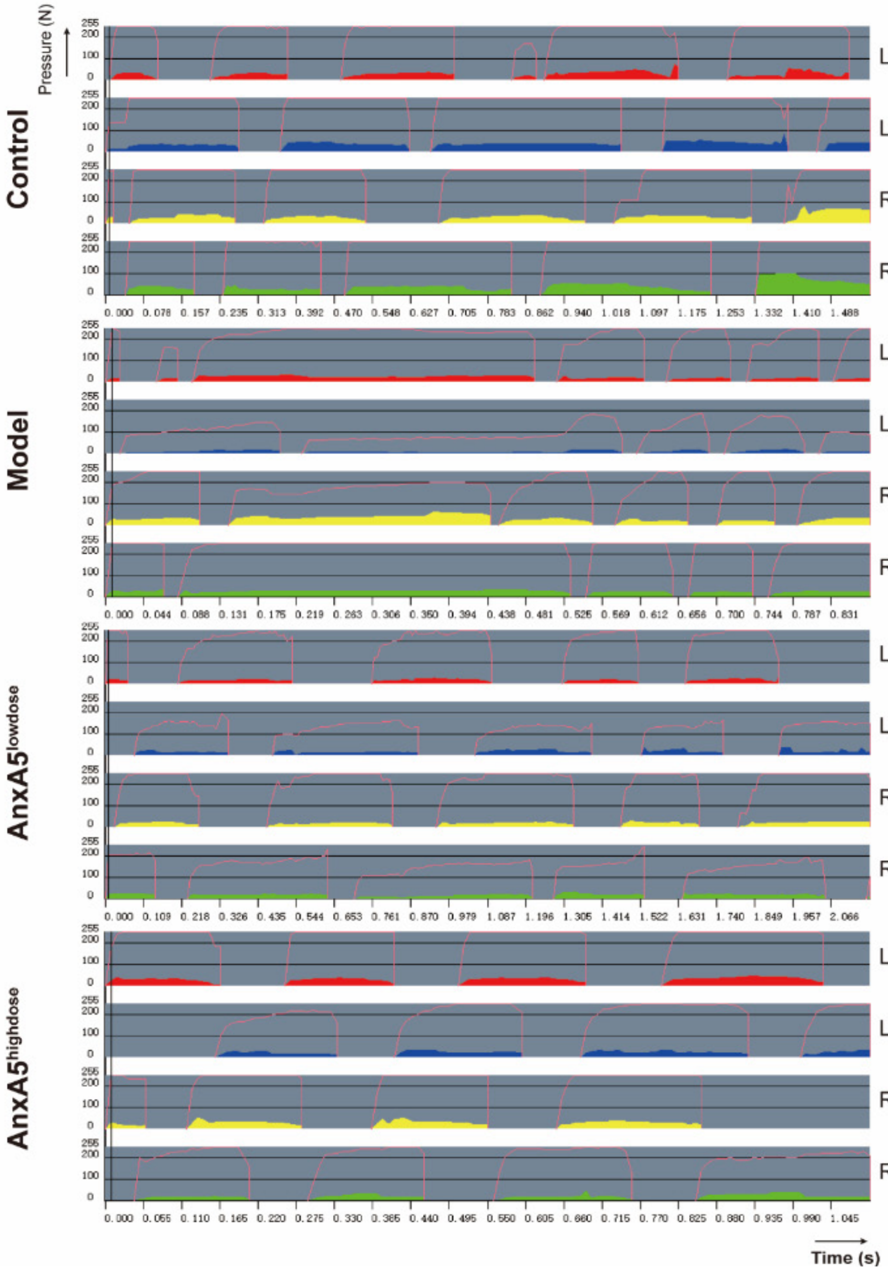

Supplementary Figure 3. Representative gait pressure analysis for RF, RH, LF, and LH in different groups.

Supplementary Table1. Gene primers used in the article.

Mouse *Il1b*-F	GAAATGCCACCTTTTGACAGTG
Mouse *Il1b*-R	TGGATGCTCTCATCAGGACAG
Mouse *Il6*-F	CTGCAAGAGACTTCCATCCAG
Mouse *Il6*-R	AGTGGTATAGACAGGTCTGTTGG
Mouse *Tnfa*-F	CAGGCGGTGCCTATGTCTC
Mouse *Tnfa*-R	CGATCACCCCGAAGTTCAGTAG
Mouse *Inos*-F	ACATCGACCCGTCCACAGTAT
Mouse *Inos*-R	CAGAGGGGTAGGCTTGTCTC
Mouse *Arg*-F	CTCCAAGCCAAAGTCCTTAGAG
Mouse *Arg*-R	GGAGCTGTCATTAGGGACATCA
Mouse *Il10*-F	GCCCTTTGCTATGGTGTC
Mouse *Il10*-R	TCTCCCTGGTTTCTCTTCC
Mouse *Tgfb*-F	CCAGATCCTGTCCAAACTAAGG
Mouse *Tgfb*-R	CTCTTTAGCATAGTAGTCCGCT
Mouse *Gapdh*-F	ATGGTGAAGGTCGGTGTGAA
Mouse *Gapdh*-R	TGAGTGGAGTCATACTGGAACA
Mouse *Cxcl10*-F	CAACTGCATCCATATCGATGAC
Mouse *Cxcl10*-R	GATTCCGGATTCAGACATCTCT
Mouse *Ccl5*-F	GTATTTCTACACCAGCAGCAAG
Mouse *Ccl5*-R	TCTTGAACCCACTTCTTCTCTG
Mouse *Cox2*-F	GCAAATTGCTGGCAGGGTTG
Mouse *Cox2*-R	CAGCATAAAGCGTTTGCGGTAC
Mouse *Cxcl11*-F	AACAGGAAGGTCACAGCCATAGC
Mouse *Cxcl11*-R	CAACTTTGTCGCAGCCGTTACTC
Mouse *Il12b*-F	GGGAGCTGGAGAAAGACGTT
Mouse *Il12b*-R	GAGCCTATGACTCCATGTCTCTG
Mouse *Il18*-F	AGACAGTGAAGTAAGAGGACTGG
Mouse *Il18*-R	TTCAGGTGGATCCATTTCCTCA
Mouse *Myd88*-F	GCGAGCTAATTGAGAAAAGGTGTC
Mouse *Myd88*-R	AAGTCCTTCTTCATCGCCTTGT
Mouse *Tlr1*-F	GGCTGCTCTTATTTCTTACGTG
Mouse *Tlr1*-R	TGAAGGAATTCCACGTTGTTTC
Mouse *Tlr2*-F	CCGAAACCTCAGACAAAGCG
Mouse *Tlr2*-R	CCCAGAAGCATCACATGACAGA
Mouse *Tlr3*-F	TCTCTGGGCTGAAGTGGACAAATC
Mouse *Tlr3*-R	AGAAGGAACCGTTGCCGACATC
Mouse *Tlr4*-F	TTGAATCCCTGCATAGAGGTAGTT
Mouse *Tlr4*-R	TGGTTGAAGAAGGAATGTCATCAG
Mouse *Tlr8*-F	TGGCTCACCATTTGTTTTACTG
Mouse *Tlr8*-R	AAAGGAGAACGTTTTTGTCTCG
Mouse *Adamts5*-F	AATGGGTTCCCAAATATGCAGGTGT
Mouse *Adamts5*-R	GTCCCATCCGTAACCTTTGGAGA
Mouse *Mmp3*-F	ACATGGAGACTTTGTCCCTTTTG
Mouse *Mmp3*-R	TTGGCTGAGTGGTAGAGTCCC
Mouse *Mmp9*-F	CTGGACAGCCAGACACTAAAG
Mouse *Mmp9*-F	CTCGCGGCAAGTCTTCAGAG
Mouse *Mmp13*-F	CTTCTTCTTGTTGAGCTGGACTC
Mouse *Mmp13*-F	CTGTGGAGGTCACTGTAGACT
